# Methicillin-Susceptible *Staphylococcus aureus* as a Predominantly Healthcare-Associated Pathogen: A Possible Reversal of Roles?

**DOI:** 10.1371/journal.pone.0018217

**Published:** 2011-04-13

**Authors:** Michael Z. David, Susan Boyle-Vavra, Diana L. Zychowski, Robert S. Daum

**Affiliations:** 1 Department of Pediatrics, The University of Chicago, Chicago, Illinois, United States of America; 2 Department of Medicine, The University of Chicago, Chicago, Illinois, United States of America; University of California, San Francisco, United States of America

## Abstract

**Background:**

Methicillin-resistant *Staphylococcus aureus* (MRSA) strains have become common causes of skin and soft tissue infections (SSTI) among previously healthy people, a role of methicillin-susceptible (MSSA) isolates before the mid-1990s. We hypothesized that, as MRSA infections became more common among *S. aureus* infections in the community, perhaps MSSA infections had become more important as a cause of healthcare-associated infection.

**Methods:**

We compared patients, including children and adults, with MRSA and MSSA infections at the University of Chicago Medical Center (UCMC) from all clinical units from July 1, 2004-June 30, 2005; we also compared the genotypes of the MRSA and MSSA infecting bacterial strains.

**Results:**

Compared with MRSA patients, MSSA patients were more likely on bivariate analysis to have bacteremia, endocarditis, or sepsis (*p* = 0.03), to be an adult (*p* = 0.005), to be in the intensive care unit (21.9% vs. 15.6%) or another inpatient unit (45.6% vs. 40.7%) at the time of culture. MRSA (346/545) and MSSA (76/114) patients did not differ significantly in the proportion classified as HA-*S. aureus* by the CDC CA-MRSA definition (*p* = 0.5). The genetic backgrounds of MRSA and MSSA multilocus sequence type (ST) 1, ST5, ST8, ST30, and ST59 comprised in combination 94.5% of MRSA isolates and 50.9% of MSSA isolates. By logistic regression, being cared for in the Emergency Department (OR 4.6, CI 1.5-14.0, *p* = 0.008) was associated with MRSA infection.

**Conclusion:**

Patients with MSSA at UCMC have characteristics consistent with a health-care-associated infection more often than do patients with MRSA; a possible role reversal has occurred for MSSA and MRSA strains. Clinical MSSA and MRSA strains shared genotype backgrounds.

## Introduction

*Staphylococcus aureus* is among the most common pathogens affecting human beings. It is a common cause of skin and soft tissue infections (SSTIs), bloodstream infections, osteomyelitis, septic arthritis, and device-related infections. Approximately 25-40% of people are asymptomatically colonized with *S. aureus* [1]. The epidemiology of *S. aureus* changed at the end of the twentieth century with the emergence of new strains, most often methicillin-resistant (MRSA), that have circulated in the general population. The new strains, known as community-associated (CA-) MRSA, have been dominated by a single genetic background, USA300, a pulsotype corresponding to ST8 by multilocus sequence typing (M. David, unpublished data) [2]. They differ from the older health care-associated (HA-) MRSA strains genotypically, in the populations they infect, and in the types of infections that they cause [3]–[5]. One distinctive feature of CA-MRSA strains is the almost universal carriage of genes for the Panton-Valentine leukocidin (PVL), a toxin rarely carried by *S. aureus* strains prior to the 1990s. CA-MRSA strains have become the most common cause of SSTIs in U.S. emergency rooms and in jails, typically among previously healthy people [6], [7]. Prior to the late 1990s, when MRSA isolates were restricted to individuals in contact with the health care system, methicillin-susceptible *S. aureus* (MSSA) strains were almost the exclusive cause of both serious and uncomplicated *S. aureus* infections among previously healthy people.

In the CA-MRSA era, the relationship between circulating MRSA and MSSA strains in the U.S. has been examined for asymptomatic colonization [8]–[15], SSTIs [9], [16], [17], and infections among children [18]. However, the relationship among MSSA and MRSA isolates causing infections of all kinds in children and adults at a single center has not been recently examined.

After noting anecdotally that few patients seemed to be presenting for care from the community with MSSA infections, we hypothesized that, as MRSA infections became more common among *S. aureus* infections in the community, perhaps MSSA infections had become less common. To explore this relationship further, we studied a representative sample of MSSA and MRSA infections at one center to compare the genotypic and phenotypic characteristics of contemporary MSSA and MRSA isolates, risk factors for MSSA and MRSA infection, and clinical syndromes caused by MSSA and MRSA isolates.

## Methods

### Setting

The study was approved by the Institutional Review Board (IRB) of the Biological Sciences Division of the University of Chicago. Consent was provided by all subjects or by their parents or guardians for their information to be stored for this study. Informed consent was obtained by telephone in a procedure approved by the IRB. The University of Chicago Medical Center (UCMC) is an academic medical center located on the south side of Chicago serving the surrounding inner-city population as well as tertiary care referral patients.

### MSSA isolate collection

The first 20 MSSA isolates identified by the Clinical Microbiology Laboratory at UCMC each month were prospectively collected from July 1, 2004 to June 30, 2005. *S. aureus* isolates from 169 unique patients (referred to as *patient-isolates* when analyzing patient-isolate dyads) were identified; isolates beyond the first obtained from each patient were excluded. Of the 169 identified isolates, 6 could not be located and 5 were found on further testing not to be *S. aureus*. Of the 158 eligible patients with an available MSSA isolate, 60 (38%) were enrolled by telephone and 87 (55%) were enrolled with a waiver of consent because they could not be reached or were deceased in December 2008-June 2009; 11 (7.0%) declined enrollment.

### MRSA isolate collection

Among 616 consecutive MRSA isolates obtained from UCMC patients in July 1, 2004-June 30, 2005 as described [19],^19^ 71 were excluded because the isolate represented asymptomatic carriage, and 545 who had a clinical infection were included in the present study. Clinical and demographic information about the patients and genotypic and phenotypic information about the isolates was tabulated as previously described [19].

### Patient data

For the 147 enrolled MSSA patients, a physician (MZD) abstracted the electronic and paper medical records at UCMC, determining age, race/ethnicity as recorded in the chart, past medical history, details of the clinical MSSA infection, and putative risk factors for exposure to MRSA. Each patient contacted for enrollment was also asked to complete a questionnaire concerning the above demographic, medical, and risk factor topics on the telephone; 65% (39/60) of those contacted by telephone completed this questionnaire. We applied the CDC case definition, used to distinguish patients with CA- from HA-MRSA infections, to assess the CA- or HA- status of MSSA isolates. The CDC case definition classifies a patient as having a CA infection if the isolate was obtained from an outpatient of from an inpatient <48 hours after admission *and lacks* the following risk factors for exposure to the health care system: hospitalization, hemodialysis, or surgery in the previous year, presence of an indwelling catheter at the time the culture is obtained [20]. Our definition differed from the CDC criteria in that we considered surgery only in the previous 6 months, rather than 12 months, to be a risk factor for HA-MRSA infection.

### Studies on MSSA isolates

Antimicrobial susceptibility testing to oxacillin, clindamycin, erythromycin, rifampin, trimethoprim-sulfamethoxazole, vancomycin, and gentamicin was performed with the Vitek 2 system (bioMérieux Vitek, Inc., Durham, NC). A D-zone test was performed for inducible clindamycin resistance for isolates found to be susceptible to clindamycin and resistant to erythromycin according to CLSI guidelines [21]. D-zone test positive isolates were considered to be resistant to clindamycin. Isolates reported to have intermediate susceptibility to an antibiotic were considered to be resistant. The MLST was determined for each MSSA isolate as described [22]. Clonal complexes were assigned using the eBURST algorithm as described. The presence of *lukF-PV* and *lukS-PV* encoding the Panton-Valentine leukocidin (PVL) toxin was performed by PCR as described [23].

### Statistical analysis

Data were tabulated for each demographic, medical history, and other patient characteristic factors. The CDC definition was used to classify MSSA and MRSA isolates as CA- or HA-*S. aureus* [20]. The risk factor data were compared for MSSA and MRSA using χ-square or Fisher Exact for dichotomous variables, or Student's *t*-test for continuous variables. Logistic regression models were developed to test the independent association of all patient and clinical variables with *p* < 0.05 on univariate analysis (Stata, v. 10, Statacorp, College Station, TX).

## Results

Among the 147 MSSA isolates from enrolled patients, 33 were excluded when they were determined to be colonizing and not from a site of infection; 114 from clinical infections were analyzed further. In the same period at UCMC, there were 545 MRSA isolates from different patients with infections; 71 isolates representing asymptomatic colonization were excluded [19].

Patients with an MSSA isolate were more likely to have private insurance than MRSA patients (*p* = 0.001). The racial/ethnic make-up of the MSSA and MRSA patient groups differed (*p* < 0.001); the MRSA group included a higher percent of African Americans than the MSSA group (74.5% vs. 46.9%). Patients with an MSSA isolate were more like to be an adult (75%) than were MRSA patients (60.6%) (*p* = 0.005). The most common MSSA infectious syndromes were SSTI (47.4%), bacteremia, endocarditis, or sepsis (19.3%), and osteomyelitis or septic arthritis (9.7%). Compared with MRSA patients, MSSA patients were more likely to have bacteremia, endocarditis, or sepsis (*p* = 0.03). Patients with a MRSA isolate were more likely to have an SSTI than MSSA patients (*p* < 0.001) (Table 1).

**Table 1:** Demographic and clinical characteristics of patients with MSSA and MRSA infections.

	MRSA, No. of patients, *n* = 545 (%)	MSSA, No. of patients, *n* = 114 (%)	*p*-value
**Clinical syndrome**			
Bacteremia, endocarditis, or sepsis	63 (11.6)	22 (19.3)	**0.03**
Osteomyelitis or septic arthritis	33 (6.1)	11 (9.7)	0.2
Pneumonia	46 (8.4)	8 (7.0)	0.6
Skin and soft tissue infection	354 (65.0)	54 (47.4)	**<0.001**
Urinary tract infection	22 (4.0)	2 (1.8)	0.4
Other*	27 (5.0)	17 (14.9)	**<0.001**
**Type of Infection**			
Invasive	175 (32.1)	44 (38.6)	0.2
Non-Invasive	370 (67.9)	70 (61.4)	–
**Age Group**			
Pediatric (<18.0 years)	215 (39.5)	29 (25)	**0.005**
Adult	330 (60.6)	85 (75)	–
**Gender**			
Male	268 (49.2)	56 (49.1)	1.0
Female	277 (50.8)	58 (50.9)	–
**Race**			
African American	406 (74.5)	53 (46.9)	**<0.001**
Caucasian	84 (15.4)	35 (31.0)	–
Latino	11 (2.0)	8 (7.1)	–
Native American	3 (0.6)	1 (0.9)	–
Unknown	41 (7.3)	17 (14.2)	–
**Type of insurance**			
Public assistance	378 (69.4)	68 (59.7)	**0.04**
Private	132 (24.2)	45 (39.5)	**0.001**
Uninsured	19 (3.5)	1 (0.9)	0.2
Unknown	16 (2.9)	0 (0)	–

*Includes abdominal abscess, toxic shock syndrome, cholecystitis, conjunctivitis, peritonitis, empyema, neurosurgical device infection, uncertain site of culture, and upper respiratory infection.

Abbreviations: *MRSA*, methicillin-resistant *Staphylococcus aureus*; *MSSA*, methicillin-susceptible *Staphylococcus aureus*.

MSSA isolates were more often obtained from inpatients more than 48 hours after hospital admission than were MRSA (26.3% vs. 19.5%), although the difference was not significant (*p* = 0.1). There was no significant difference in the percent of MRSA and MSSA patients who had surgery in the previous 6 months, a hospital or long-term care facility stay or hemodialysis in the previous year, or an indwelling catheter at the time of culture. There was no significant difference in the percent of MSSA and MRSA patients, by self-report (*p* = 0.3) or laboratory report at UCMC (after 1993) (*p* = 0.4), who had MRSA isolated in the past (Table 2).

**Table 2:** Presence of CDC risk factors for HA-MRSA* among patients with MSSA and MRSA infections.

	MRSA, No. of patients, *n* = 545 (%)	MSSA, No. of patients, *n* = 114 (%)	*p*-value
Inpatient culture obtained >48 hours after admission	106 (19.5)	30 (26.3)	0.1
Hospital stay, past year	225/458 (49.1)	57 (50.0)	0.9
Surgery, past 6 months	209/486 (43.0)	43 (37.7)	0.3
Hemodialysis, past year	35 (6.4)	9 (8.0)	0.6
Indwelling catheter	69 (12.7)	16 (14.0)	0.7
Previous MRSA isolation			
Laboratory	58 (10.6)	15 (13.2)	0.4
report			
Self-report only	9/281 (3.2)	1 (0.9)	0.3
Lived in long-term care facility, past year	16/290 (5.5)	2 (1.8)	0.1

*Absence of these risk factors comprise the CDC case definition for community-associated MRSA infections. Denominators for HA-MRSA risk factors exclude those interviewed patients who answered that that they did not know information requested of them and those patients about whom risk factor information could not be determined from chart review. For all 659 patients it was determined whether MRSA had been isolated from them at UCH since 1994, but for 295 patients, it could not be determined if MRSA had been isolated from them at another health care facility. The information regarding a stay in a long-term care facility was determined only for those patients lacking another health-care risk factor.

Abbreviations: *HA-*, health care associated; *MRSA*, methicillin-resistant *Staphylococcus aureus*; *MSSA*, methicillin-susceptible *Staphylococcus aureus*.

MRSA (346/545) and MSSA (76/114) patients did not differ significantly in the proportion that would be classified as HA-*S. aureus* by the CDC CA-MRSA definition (*p* = 0.5).

MSSA and MRSA patients did not differ significantly in the likelihood that they had a comorbid condition, including an immunocompromised state, diabetes mellitus, cystic fibrosis, cancer, or HIV infection. Patients with MSSA were more likely to be transplant recipients than MRSA patients (*p* = 0.002). MRSA patients were more likely than MSSA patients to have been in jail (*p* = 0.005). MRSA patients lived in larger households than MSSA patients (*p* = 0.047) (Table 3).

**Table 3:** Additional Risk Factors and Putative Risk Factors for Exposure to MRSA.

	MRSA, No. of patients, *n* = 545 (%)	MSSA, No. of patients, *n* = 114 (%)	*p*-value
Antibiotic use in past 6 months	229 (42.1)	57 (50)	0.1
Immunocompromised*	80 (14.7)	22 (19)	0.2
Diabetes	103 (18.9)	24 (21)	0.6
Cancer	87 (16.0)	22 (19)	0.4
Transplant	12 (2.2)	9 (8.0)	0.002
Implant, hardware or other foreign body	88 (16.1)	20 (18)	0.7
HIV-infected	13 (2.4)	4 (3.5)	0.5
Attend daycare	31/215 (14.4)	3/29 (10)	0.8
Work in prison or jail	15 (2.8)	2 (1.8)	0.8
Been in prison or jail	41 (7.5)	1 (0.9)	0.005
Travel in previous 6 months	56 (10.3)	9 (7.9)	0.4
Intravenous drug use	29 (5.3)	2 (1.8)	0.1
Household healthcare contact†	65/303 (21.5)	10/49 (20)	0.9
Household hospitalized contact‡	67/295 (22.7)	7/47 (15)	0.2
Number people in household, mean ± s. d.¶	3.66±2.16	3.15±1.51	0.047
Number bedrooms in household mean ± s. d.∫	2.88±1.28	2.87±0.89	1.0
Number persons/bedroom in household, mean ± s. d.¶	0.89±0.03	1.02±0.07	0.1

*Patients either had an inborn immunodeficiency or were infected with HIV at the time of culture or that they were taking immunosuppressive drugs at the time of culture. Patients with diabetes mellitus, liver disease, cancer, or a rheumatologic disease were not considered to be immunocompromised in the absence of immunosuppressive drug therapy.

†Denominators indicated are the number of patients interviewed who answered the relevant questions.

‡For MRSA *n* = 424; for MSSA, *n* = 79.

∫For MRSA *n* = 277; for MSSA, *n* = 45.

¶For MRSA, *n* = 276; for MSSA, *n* = 45.

Abbreviations: *MRSA*, methicillin-resistant *Staphylococcus aureus*; *MSSA*, methicillin-susceptible *Staphylococcus aureus*.

**Figure 1:**
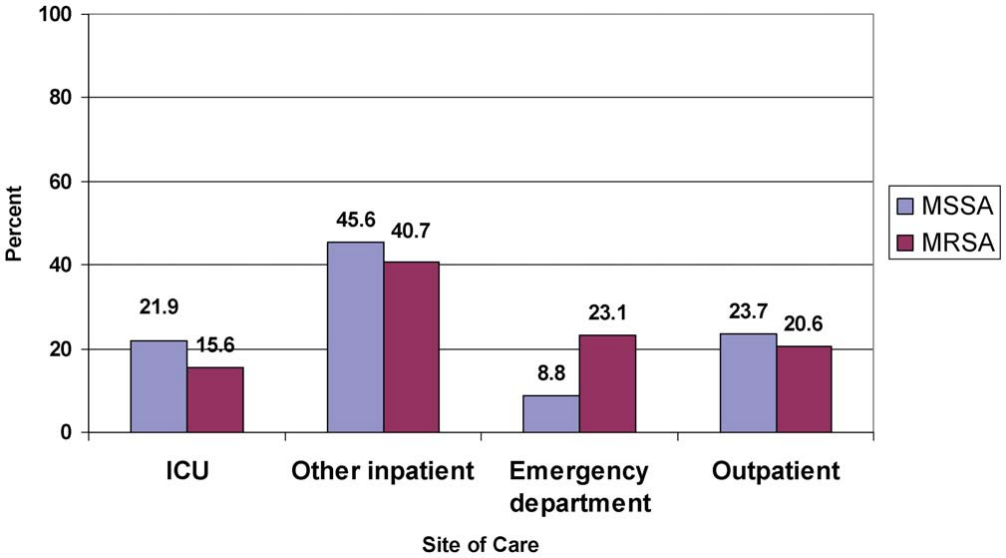
Percent of patients with MRSA and MSSA infections who had cultures obtained in various sites of care; emergency department patients include only those cultured and not admitted to the hospital.

At the time of culture, MSSA patients were more likely than MRSA patients to be in the intensive care unit (21.9% vs. 15.6%) or another inpatient unit (45.6% vs. 40.7%) whereas MRSA patients were more likely to be in the emergency department (23.1% vs. 8.8%) (Figure 1).

**Figure 2:**
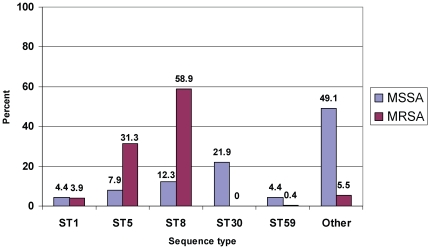
Percent of MSSA and MRSA isolates from UCMC, July 1, 2004-June 30, 2005, belonging to ST1, ST5, ST8, ST30, ST59, and other genetic backgrounds.

The MSSA isolates were more polyclonal than were the MRSA isolates. Among the MSSA isolates, there were 24 STs (representing 12 clonal clusters [CC] and 2 STs that did not belong to a defined CC). Among the MRSA isolates, there were 11 STs (in 6 CC). There was substantial overlap in the ST/CC repertoire of the MRSA and MSSA isolates. ST1, ST5, ST8, ST30, and ST59, all common genetic backgrounds of clinical MRSA isolates in the U.S. and other parts of the world, comprised, in aggregate, 94.5% of the MRSA isolates; among the MSSA isolates, these STs comprised 50.9% of the aggregate MSSA isolates (Figure 2, Table 4). Of MRSA isolates, 33% (180) carried SCC*mec* type II and 67.7% (358) carried SCC*mec* type IV, and 1.3% (7) did not SCC*mec* elements typable by the routine PCR assays used.

**Table 4:** MLST of MRSA and MSSA isolates causing infections at UCMC.

	MRSA		MSSA	
	Number (*n* = 545)	Percent	Number (*n* = 114)	Percent
Clonal complex type/MLST				
*Clonal complex 1* *				
1	21	3.9	5	4.4
188	0	0	1	0.9
573	0	0	1	0.9
*Clonal complex 5*				
5	170	31.3	9	7.9
5slv†	1	0.2	1	0.9
105	3	0.6	0	0
231	14	2.6	0	0
*Clonal complex 8*				
8	321	58.9	14	12.3
8slv†	2	0.3	0	0
72	1	0.2	5	4.4
1181	0	0	1	0.9
*Clonal complex 9*				
9	0	0	1	0.9
109	0	0	4	3.5
*Clonal complex 12*				
12	0	0	1	0.9
12slv†	0	0	1	0.9
*Clonal complex 15*				
15	0	0	8	7.0
582	0	0	1	0.9
*Clonal complex 20*				
20	0	0	3	2.6
*Clonal complex 22*				
22	6	1.1	0	0
*Clonal complex 30*				
30	0	0	25	21.9
30slv†	0	0	1	0.9
36	3	0.6	0	0
39	0	0	1	0.9
*Clonal complex 59*				
59	2	0.4	5	4.4
*Clonal complex 97*				
97	0	0	2	1.8
*Clonal complex 121*				
121	0	0	1	0.9
*Clonal complex 182*				
182slv†	0	0	1	0.9
*Singletons*				
580	0	0	1	0.9
1159	0	0	1	0.9

*CC1 was subsumed by the CC15 by the MLST administrators.

†*slv*, single locus variant.

Abbreviations: *MLST*, multilocus sequence typing; *MRSA*, methicillin-resistant *Staphylococcus aureus*; *MSSA*, methicillin-susceptible *Staphylococcus aureus*.

PVL gene carriage (PVL+) was common among the MRSA isolates; significantly fewer MSSA isolates were PVL+ (58.2% vs. 7.0%, *p* < 0.001). Among the 8 PVL+ MSSA isolates, 6 were ST8, 1 was ST1 and 1 was ST121. The proportion of PVL+ isolates did not differ significantly among the ST8 MSSA (6/8, 75%) and ST8 MRSA (290/321, 90.3%) backgrounds (*p* = 0.2). The syndromes caused by the 6 ST8, PVL+ MSSA isolates were uncomplicated SSTIs in 3 and an abscess associated with a transcutaneous gastric tube, a surgical wound infection, and a central venous catheter-associated bacteremia in 1 patient each. The ST121, PVL+ MSSA isolate was obtained from a patient with septic arthritis and pyomyositis; the ST1 PVL+ MSSA isolate came from a patient with an uncomplicated SSTI. Among the 44 invasive MSSA infections, just 2 (4.6%) were caused by PVL+ strains.

In Model 1, including a history of incarceration as a covariate, African American race (OR 0.37, CI 0.15-0.91, *p* = 0.03) and a history of having ever been incarcerated (7.9 CI 1.7–36.9, *p* = 0.008) were independently associated with a MRSA infection. Care in the Emergency Department (OR 3.4 compared with patients from inpatient non-ICU units, CI 0.84–14.0, *p* = 0.09) and an increasing number of people in the household (OR 1.3, CI 0.98–1.7, *p* = 0.07) trended toward significance among MRSA patients. Only 184 patients were included in Model 1 because data were available about history of incarceration from only 195 (48 MSSA and 147 MRSA) patients, and 11 of these lacked data on other variables in the model (Table 5a). In Model 2, omitting the history of incarceration (*n* = 455), only care in the Emergency Department (OR 4.6 compared with patients from inpatient non-ICU units, CI 1.5–14.0, *p* = 0.008) was significantly associated with MRSA infection. Being a transplant patient trended toward a significant association with MSSA infection (OR 0.40, CI 0.13–1.1, *p* = 0.08) (Table 5b).

**Table 5:** (a) Logistic regression Model 1. including variables demonstrating significant association with MRSA infection on bivariate analysis (*n* = 184); (b) Logistic regression Model 2. same as Model 1. *excluding* variable for ever been incarcerated (*n* = 455).

Characteristic	OR (95% CI)	*p*-value
**Patient characteristics**		
Non-African American race*	0.37 (0.15–0.91)	0.03
Pediatric Age Group	0.86 (0.29–2.6)	0.8
Public Insurance or Uninsured†	1.9 (0.77–4.6)	0.2
Number of people in household‡	1.3 (0.98–1.7)	0.07
Ever been incarcerated	7.9 (1.7–36.9)	0.008
Transplant patient	0.48 (0.11–2.1)	0.3
**Other factors**		
Location of care		
Inpatient (non-ICU)	ref.	–
ICU	1.6 (0.48–5.4)	0.5
Emergency Department	3.4 (0.84–14.0)	0.09
Outpatient clinic	0.46 (0.15–1.4)	0.2
SSTI	1.2 (0.44–3.0)	0.8
5b. Model 2		
**Patient characteristics**		
Non-African American race∫	0.61 (0.34–1.1)	0.1
Pediatric Age Group	0.95 (0.47–1.9)	0.9
Public Insurance or Uninsured¶	1.4 (0.79–2.5)	0.3
Number of people in household‡	1.08 (0.92–1.3)	0.3
Transplant patient	0.40 (0.13–1.1)	0.08
**Other factors**		
Location of care		
Inpatient (non-ICU)	ref.	–
ICU	1.3 (0.58–2.7)	0.6
Emergency Department	4.6 (1.5–14.0)	0.008
Outpatient clinic	1.2 (0.47–1.9)	0.7
SSTI	1.3 (0.74–2.4)	0.3

*African American (*n* = 139) compared with all others of known race (*n* = 45).

†Public Insurance or uninsured (*n* = 131) compared with privately insured (*n* = 53).

‡Indicates odds ratio for every additional person in the household.

∫African American (*n* = 353) compared with all others of known race (*n* = 102).

¶Public Insurance or uninsured (*n* = 333) compared with privately insured (*n* = 122).

Abbreviations: *SSTI*, skin or soft tissue infection.

## Discussion

Our data yield three major conclusions. First, MSSA has, by several criteria become a predominantly health-care-associated pathogen among patients at UCMC. Moreover, MRSA patients are no longer predominantly patients with recent exposure to the health care setting. Thus, a possible role reversal has occurred for MSSA and MRSA strains; before the mid-1990s, MRSA was almost exclusively a health care-associated pathogen, and *S. aureus* infections in the community were nearly always MSSA. We found that MSSA patients were more likely to have bacteremia, endocarditis, or sepsis, to be transplant patients, to be adults, and to be in an intensive care unit than MRSA patients. MSSA patients were also less likely than MRSA patients to have an SSTI or to be treated in the emergency department and released. MSSA and MRSA patients had a similar likelihood of having an invasive infection. MRSA infections were not more likely than MSSA infections to occur among patients with a number of chronic diseases.

The second conclusion is that there was considerable overlap in the MLST genotypes of MRSA and MSSA strains. Although the MSSA strains we studied were more polyclonal than the MRSA strains, as others have noted [24], we found that both the MRSA and MSSA isolates from patients seeking care for infections mostly fell into 5 STs: ST1, ST5, ST8, ST30, and ST59, all common MRSA genetic backgrounds in various regions of the world. Thus, despite the emergence of novel CA-MRSA strains during the previous decade in Chicago, the genotypic backgrounds among MSSA strains causing infections at our center maintained substantial genotypic overlap with MRSA strains.

Third, we have demonstrated that MSSA strains causing clinical infections rarely carry genes for the PVL toxin (7.0%), in contrast to MRSA strains (58.2%) even in the era of epidemic CA-MRSA. The low prevalence of PVL gene carriage in MSSA strains is consistent with reports from European centers prior to the emergence of CA-MRSA [24]. Further research is needed to determine why PVL toxin genes are almost universally carried by CA-MRSA strains but remain rare among MSSA and HA-MRSA strains; these genes presumably impart either a survival advantage or they represent virulence determinants in these strains that became widespread in the late 1990s.

Most MSSA strains that were PVL+ were ST8, the same ST of strains with the USA300 pulsotype, and most ST8 strains were obtained from uncomplicated SSTIs, the most common type of infection caused by CA-MRSA in many studies in the U.S. These data indicate an association between ST8 *S. aureus* strains, regardless of the methicillin resistance phenotype, and presence of an SSTI.

Others have also found that MSSA strains commonly cause invasive infections.^4, 18^ The importance of ST8 (consistent with USA300) among MSSA invasive disease isolates, however, is unclear. We found that ST8 MSSA isolates were infrequently responsible for invasive disease although others have established an important role for ST8 isolates among invasive infections in children [17], [25]. We also found few invasive infections caused by PVL+ MSSA strains. The results of these studies may differ because we also studied adults or because of temporal or geographic variation in the MRSA epidemic.

Our study suggests that patients living in poverty and underserved populations are at higher risk for a MRSA infection, but not an MSSA infection. Private insurance was more common among MSSA than MRSA patients, and MRSA patients were more commonly treated in the emergency department. This is consistent with our finding that MSSA infections reflect epidemiologic characteristics of nosocomial or health-care associated infections. Our patients with MSSA isolates were not as likely as patients with CA-MRSA infections to be previously healthy and presenting for care from the community.

Other studies have compared MRSA and MSSA patients, but they have been limited by a lack of genotyping data, or limited to isolates obtained from skin infections and/or CA-*S. aureus* infections (defined by various criteria) [4], [16]–[18], [26], [27]. McCarthy *et al.* examined MRSA and MSSA infections among military veterans in Atlanta in 2007–2008 and found that MRSA patients were more likely to have had a previous MRSA infection and to have had a stay in a long-term care facility in the previous year, but less likely to have had a biopsy in the previous year [27]. We did not find the same distinctions, perhaps because of differences in the populations studied.

There are limitations to our study. We examined *S. aureus* infections at one medical center in one city. The MSSA patients studied were not a complete cohort, but the 114 patients and isolates that we examined were a carefully designed sample that is likely representative of all MSSA infections at our institution.

In conclusion, MSSA has reversed roles with MRSA. In the era of epidemic CA-MRSA infections, MSSA isolates have assumed the role of a nosocomial pathogen among the debilitated, while CA-MRSA strains continue to predominate among patients in our inner-city community.
